# Genetic Diversity of Potato Leafroll Virus (Polerovirus *PLRV*) Is Shaped by Variant Displacements and Selective Pressures Imposed by Aphid and Tuber Transmission Routes

**DOI:** 10.3390/v17101294

**Published:** 2025-09-24

**Authors:** Graham H. Cowan, Catherine Thomson, Emma Back, Lesley Torrance, Christophe Lacomme, Eugene V. Ryabov

**Affiliations:** 1 Cell and Molecular Sciences Department, The James Hutton Institute, Dundee DD2 5DA, UK; graham.cowan@hutton.ac.uk (G.H.C.); lesley.torrance@hutton.ac.uk (L.T.); 2 Science and Advice for Scottish Agriculture, Edinburgh EH12 9FJ, UK; catherine.thomson@sasa.gov.scot (C.T.); emma.back@sasa.gov.scot (E.B.); christophe.lacomme@sasa.gov.scot (C.L.)

**Keywords:** RNA virus, *Polerovirus PLRV*, *Solemoviridae*, *Solanum tuberosum*, virus evolution, high-throughput sequencing

## Abstract

Potato leafroll virus (PLRV, species *Polerovirus PLRV*) is a major pathogen affecting potatoes worldwide. Since 2018, PLRV incidence has increased in Scottish potato crops. Deep sequencing of PLRV in Scottish potato plants revealed the prevalence of a novel PLRV type which became predominant in 2023, displacing the phylogenetically distinct variants that have been present in the region since at least 1989. Analysis of the infection dynamics of the cDNA clone-derived PLRV isolates in potato plants indicated that the novel PLRV may accumulate to higher levels compared to the historic one. Analysis of the genetic diversity of PLRV in early and late field generations (FGs) of seed potatoes showed a significantly reduced genetic diversity of the PLRV structural genes in the early FGs compared to the late FGs, while divergency of the non-structural genes remained similar across all FGs. Considering that late FGs are more likely to be infected with PLRV via tuber transmission, and early FGs via aphid transmission, these findings suggest that aphid transmission imposes a genetic bottleneck on the structural genes of PLRV, but not on its non-structural genes.

## 1. Introduction

Potato leafroll virus (PLRV) is a positive-strand RNA virus which belongs to the species *Polerovirus PLRV* within the family *Solemoviridae* [1,2,3,4]. PLRV is a major pathogen of potato (*Solanum tuberosum* L.) which is present in all potato-growing regions worldwide and causes significant losses in yield and quality of potato tubers [5,6,7]. PLRV has 5.8 kb positive-strand genomic RNA which contains a VPg protein covalently linked to the 5′ terminus. The PLRV genome contains eight open reading frames (ORFs), some of which overlap. The viral RNA replicase is encoded by ORF1 and ORF2, the structural genes are encoded by ORF3 and ORF5, the suppressor of RNA silencing and phloem movement protein are encoded by ORF0 and ORF4, respectively. Expression of PLRV ORFs involves translational frameshift, readthrough, leaky scanning, and ribosome scanning strategies [5]. The 5′ proximal ORFs 0, 1, 2, and 8 are expressed from the genomic RNA, while expression of the 3′ proximal ORFs 3a, 3, 4, 6, and 7 occurs from two subgenomic (sg) RNAs sharing the same 3′ as the genomic RNA [4,5]. The larger sgRNA is required for translation of ORF3, ORF3a, ORF4, and ORF5. The C-terminal 125 amino acid (AA) portion of the ORF5-encoded protein could be expressed as the ORF7 protein from the shorter sgRNA [5].

PLRV forms non-enveloped icosahedral virus particles, approximately 24 nm in diameter, composed of 180 structural protein subunits, the majority of which are coat protein (CP) subunits encoded by ORF3. During PLRV infection, the readthrough protein (RTP) which contains the ORF3-encoded CP fused to the ORF5-encoded readthrough domain (RTD) is produced as a result of translational readthrough of the amber codon of ORF3 [4]. The RTP subunits are incorporated into PLRV virus particles as a minor structural protein, with the N-terminal CP part interacting with other CP subunits to form the virion shell, with the RTD protruding from the surface of the virion [8]. The N-terminal part of RTD (^N^RTD) encoded by ORF5 plays a key role in aphid transmission [9]. The C-terminal part of the RTD (^C^RTD) is proteolytically processed at multiple sites and several truncated RTD variants are included in the PLRV particle [10].

Like other members of the genus *Polerovirus*, PLRV is a phloem-limited virus, which replicates and moves only in the cells of the phloem. The systemic movement of PLRV within plants requires structural proteins encoded by ORF3 and ORF5, as well as movement protein encoded by ORF4 [4,5]. The systemic movement through the phloem of potato plants also allows PLRV infection to reach developing tubers, enabling vertical transmission of the virus to the next vegetative generation. The virus is transmitted between plants by aphids, principally by *Myzus persicae*, in a non-replicative persistent manner [4,5]. Virion assembly is essential for both phloem movement and the aphid transmission of PLRV. Both the CP and minor RTD are required for the systemic movement of PLRV through the phloem and aphid transmission, which involves transition of the virus particles through midgut and salivary gland epithelial cells and circulation in the hemolymph [9,11]. Interactions with phloem and aphid factors may exert different selective pressures on the structural proteins of PLRV; the genetic composition of PLRV populations may be affected by aphid and tuber transmissions.

Although PLRV incidence in Scottish potatoes remains low, PLRV incidence has increased since 2018 [12], which could be explained by the higher abundance of aphid vectors of the virus due to the recent ban on the pesticides used for aphid control [13], or other environmental or cultural factors. It is also known that virus epidemics could be a result of the introduction and spread of a novel virus variant(s) [14]. To test this possibility, we analyzed the genetic diversity of PLRV in potato crops using high-throughput sequencing. Our analysis of symptomatic seed potato samples collected across Scotland in 2023 showed the prevalence of a single novel phylogroup of PLRV which apparently had displaced isolates of a different phylogroup present in Scotland since at least 1989 [2]. We also investigated the hypothesis that genetic changes in the PLRV populations might have been a result of the virus’s adaptation to the aphid and tuber transmission routes by deep sequencing of PLRV populations from different field generations (FGs) of seed potatoes, which might have acquired PLRV predominantly either from the aphid vectors or vertically from infected tubers. Further, we engineered an infectious cDNA clone of the contemporary Scottish PLRV variant and compared infection dynamics of the clone-derived Scottish PLRV isolates from both phylogroups in potato plants. While this approach does not represent a natural mode of infection, preliminary data suggest a higher accumulation of the PLRV isolate of the currently prevalent phylogroup. Further analyses are needed to conclude whether the prevalent PLRV type has a selective advantage over historic PLRV isolates in a natural infection context.

## 2. Materials and Methods

### 2.1. PLRV Samples

To analyze the genetic diversity of PLRV in seed potato crops, leaf samples from individual potato plants (*n* = 889) of 2nd to 6th field generations (FG2 to FG6) showing putative symptoms of PLRV infection were collected across Scottish seed potato-growing areas in the summer of 2023. Initial screening for the virus was carried out in support of the Scottish Seed Potato classification scheme by Science and Advice for Scottish Agriculture (SASA). The presence of PLRV infection was identified by ELISA tests [15]. A representative set of PLRV-infected samples from different FGs was selected for deep sequencing (Table 1). In addition, ware potato plants displaying leafroll symptoms were collected in the summer of 2024 to analyze PLRV circulating in Scotland in the following growing season. This material was used as source in the subsequent production of an infectious cDNA clone (Table 1). To further characterize isolates of the virus in potato crops from previous years, we sequenced PLRV from the potato plants which originated from PLRV-infected seed potato plants collected from seed potato fields in Scotland in 2010, which were then tuber-propagated in an insect-proof glasshouse every year until sampling (Table 1).

### 2.2. High-Throughput Sequencing of PLRV

Total RNA was extracted from potato leaf material using the NucleoSpin RNA plant kit (Macherey-Nagel, Düren, Germany). Reverse transcription was carried out using LunaScript (New England Biolabs, Ipswich, MA, USA) with PLRV-specific reverse primers PLRV-4158REV (5′-CTGAAGGATCCTGCGGTATC-3′) and PLRV-REV (5′-ACTACACAACCCTGTAAGAGGATCC-3′) to produce cDNAs for the 5′ and 3′ sections of the viral genome, respectively. Two overlapping RT-polymerase chain reaction (RT-PCR) fragments, 4.2 kb and 2.7 kb, covering the entire 5.8 kb PLRV genome were amplified with proof-reading Phusion polymerase (New England Biolabs). The 5′ section was amplified with the primers PLRV-FOR (5′-ACAAAAGAATACCAGGAGGAATTGC-3′) and PLRV-4158-REV, and the 3′ genome section was amplified with the primers PLRV-3115FOR (5′-GGAGTATAAAACACTAGGTTTCAAAG-3′) and PLRV-REV. The RT-PCR fragments were isolated from agarose gel with a Gel Extraction Kit (QIAgen, Hilden, Germany).

High-throughput sequencing was performed at the James Hutton Institute Genomic facility. The 5′ and 3′ RT-PCR fragments derived from each sample were pooled equimolarly, quality control of the amplicon pools was performed using Bioanalyzer 100 using the recommended procedure, and the final pools were diluted to 6 pM containing 20% PhiX control DNA and loaded on Illumina MiSeq (Illumina, Inc., San Diego, CA, USA) with paired-end 150 bp reads using standard parameters. Post-run fastq files were generated, samples de-multiplexed, and adapters removed onboard for each sample. Libraries of approximately 25 thousand paired-end 150 nt reads were produced for each sample. Cleaned data from each library were then used for de novo assembly of the viral contig sequences using rnaSPAdes v3.15.4 (standard k-mers: 21 nt) [16]. The de novo assembled contigs corresponding to the full-length PLRV genomic RNAs were deposited in NCBI GenBank, with accession numbers PP584529 to PP584549 and PQ047449 to PQ047454 (Table 1).

To characterize PLRV in the symptomatic samples collected in the year 2024, two overlapping PLRV RT-PCR amplicons were produced as above and then sequenced by using both the Sanger method and Oxford Nanopore technology with Eurofins Genomics Germany GmbH. The sequences were submitted to NCBI GenBank, with accession numbers PV647837 and PV647838 (Table 1).

### 2.3. Bioinformatic and Statistical Analyses

The full-length contig open reading frames (ORFs) of PLRV sequences were analyzed and compared against sequences deposited to GenBank by the BLAST search tool (version 2.17.0) [17]. The full-length sequences of PLRV genomic RNA produced in this study and all full-length genomic sequences of PLRV available in NCBI GenBank (*n* = 87) were aligned with the ClustalW [18] (Galaxy Version 2.1+galaxy1) tool, and the alignment was used to produce a maximum likelihood (ML) tree using the RAxML tool (Galaxy Version 8.2.12+galaxy1) using the Galaxy server. Bootstrap values were obtained from 100 replicates with the PHYLIP package [19]. The trees were visualized by using the Figtree tool (http://tree.bio.ed.ac.uk/software/figtree/, accessed on 9 May 2025).

Identification of codons under both stabilizing and diversifying selection was carried out by estimating the relative rates of synonymous (dS) and non-synonymous substitutions (dN) in the PLRV infecting seed potatoes in the year 2023, by using web-based Datamonkey tools (http://www.datamonkey.org, accessed on 3 July 2025) [20].

Pairwise distances between sections of de novo assembled PLRV contigs were calculated using Clustal W [18]. Statistical analyses were conducted using R Software version 4.2., and figures were produced using the package ggplot2 [21].

### 2.4. Design and Evaluation of PLRV cDNA Clones

Construction of the full-length infectious cDNA clone of a typical contemporary Scottish PLRV isolate was carried out essentially as previously [22]. Total RNA was extracted from potato plants showing PLRV symptoms sourced in July 2024 in Forfar, Scotland, and was used to produce cDNA with the Lunascript RT Supermix Kit (New England Biolabs) and reverse primer PLRV-R2 (5′-TGGAGATGCCATGCCGACCCACTACACAACCCTGTAAGAGGATCC-3′) which was complementary to the 3′ terminus of PLRV genomic RNA. The cDNA was used as a template to produce a PCR fragment using proof-reading Phusion High Fidelity DNA polymerase (New England Biolabs) and primers PLRV-R2 and PLRV-F2 (5′-GGAAGTTCATTTCATTTGGAGAGGACAAAAGAATACCAGGAGGAATTGC-3′). The PLRV amplicon was cloned into a vector pDIVA (GenBank Accession Number KX665539), based on the mini binary vector pCB [23], supplemented with a cauliflower mosaic virus (CaMV) 35 S promoter followed by a hepatitis delta virus (HDV) ribozyme and the polyadenylation signal of CaMV. The vector was amplified by inverse PCR using primers pDIVA-F (5′-CCTCTCCAAATGAAATGAACTTCC-3′) and pDIVA-R (5′-GGGTCGGCATGGCATCTCCA-3′). Ligation of the vector and PLRV cDNA amplicons was performed using the NEBuilder HiFi DNA kit (New England Biolabs, Ipswich, MA, USA); standard molecular cloning techniques were used to produce the full-length PLRV clone pDIVA-PLRV-Scot-2024. The plasmid clone was sequenced by both the Sanger method and Oxford Nanopore technology with Eurofins Genomics Germany GmbH; the sequence was submitted to NCBI GenBank, with accession number PQ868077 (Table 1). pDIVA-PLRV-Scot-2024 was transformed into Agrobacterium tumefaciens strain LB4404. Inoculation of potato plants (cv Desiree) with cDNA clone-derived PLRV was carried out using an agrobacterium culture method involving incubating the freshly cut stem section with the agrobacterium cultures with PLRV cDNA clones [22]. The plants were rooted in pots containing compost and were grown in a glasshouse with 16 h light (18 °C) and 8 h darkness (15 °C).

The levels of PLRV RNA in potato plants were quantified by RT-qPCR (reverse transcription quantitative PCR). Total RNA was extracted as above. The first strand cDNA synthesis was carried out using LunaScript (New England Biolabs) with random primers, and qPCR was carried out with a SYBR green kit with the primers qPLRV- REV (5′-GCACAGAGAATACTGCAGCCAG-3′ and qPLRV_FOR (5′-TTCAGAAATCCGACCCTCGC-3′) for the PLRV RNA target and the primers qActin_rev (5′-TGATTTGAGTCATCTTCTCACG-3′) and qActin-FOR (5′-GAGCAATTGGGATG ACATGG-3′) for the potato beta-actin mRNA target. The cycle threshold (Ct) values for PLRV were subtracted from the Ct values for the beta-actin to obtain normalized DCt values, which were used to compare loads of PLRV genomic RNA in potato tissue samples.

Phylogroup identity of PLRV in RNA samples from tuber-propagated potato plants was determined by RT-PCR using primer pairs for the region coding for the C-terminal part of RTD specific either to PLRV phylogroup N (5′-CGAAAGCCGATCTTTTAGAA-3′, and 5′-CAAGCGCTCTTCATAAGTCTGT-3′) or PLRV phylogroup O (5′-CGAAAGCCGATCTACTGGAG-3′ and 5′-TAAACGCTCTTCAACAGTTACC-3′). The amplified RT-PCR fragments were sequenced by Sanger.

For the expression of the structural proteins of PLRV of the historic and novel Scottish types tagged with the green florescent protein (GFP), the ORF3-ORF5 coding sequences were amplified by PCR from the infectious cDNA plasmid constructs (GenBank accession numbers OK245432 and PQ868077, respectively) with the primers AttB1-PLRV-P3P5-for (5′-GGGGACAAGTTTGTACAAAAAAGCAGGCTCGATGAGTACGGTCGTGGTTAAAGGAAATGTCAATGGTG-3′) and AttB2-stp-PLRV-REV (5′-GGGGACCACTTTGTACAAGAAAGCTGGGTttagttaACTACACAACCCTGTAAGAGGATCC-3′) and cloned, using the Gateway cloning approach, into the binary plant expression vector pGWB406 to produce in-frame GFP fusion for expression under the control of the CaMV 35S promoter.

## 3. Results

### 3.1. Identification of a Prevalent PLRV Phylogroup Which Became Predominant in Scottish Potatoes

The genetic diversity of PLRV across all Scottish potato-growing areas in 2023 (Table 1) was determined. For the analysis, we sampled individual potato plants of different field generations (FGs) of seed potatoes. To obtain additional sequenced data on the PLRV variants circulating in Scotland previously, we analyzed PLRV genomes in potato plants originating from the tubers collected in the field in 2010 and propagated by tuber transmission in an aphid-free glasshouse until sampling in 2018, 2019, or 2023. Furthermore, we analyzed several samples of ware potatoes in the year 2024. We produced RT-PCR amplicons covering the entire PLRV genome, which were sequenced using a high-throughput sequencing approach. The sequencing libraries were used to produce the full-length de novo assembled contigs.

Scottish seed potato production starts from in vitro propagated virus-free micro-plants to produce mini-tubers that can be grown in the field to produce progeny tubers (field generation one; FG1). The field-grown progeny tubers are harvested and planted for up to 6 consecutive years to multiply seed tuber output. PLRV could be introduced into potato plants by aphids and then transmitted via tubers. Therefore, the assumption was made that analysis of the samples collected in a single growing season from early and late FGs could give a snapshot of PLRV populations most likely transmitted by aphids in crops of earlier FGs and up to 5 previous years of tuber propagation for crops of later FGs.

**Figure 1:**
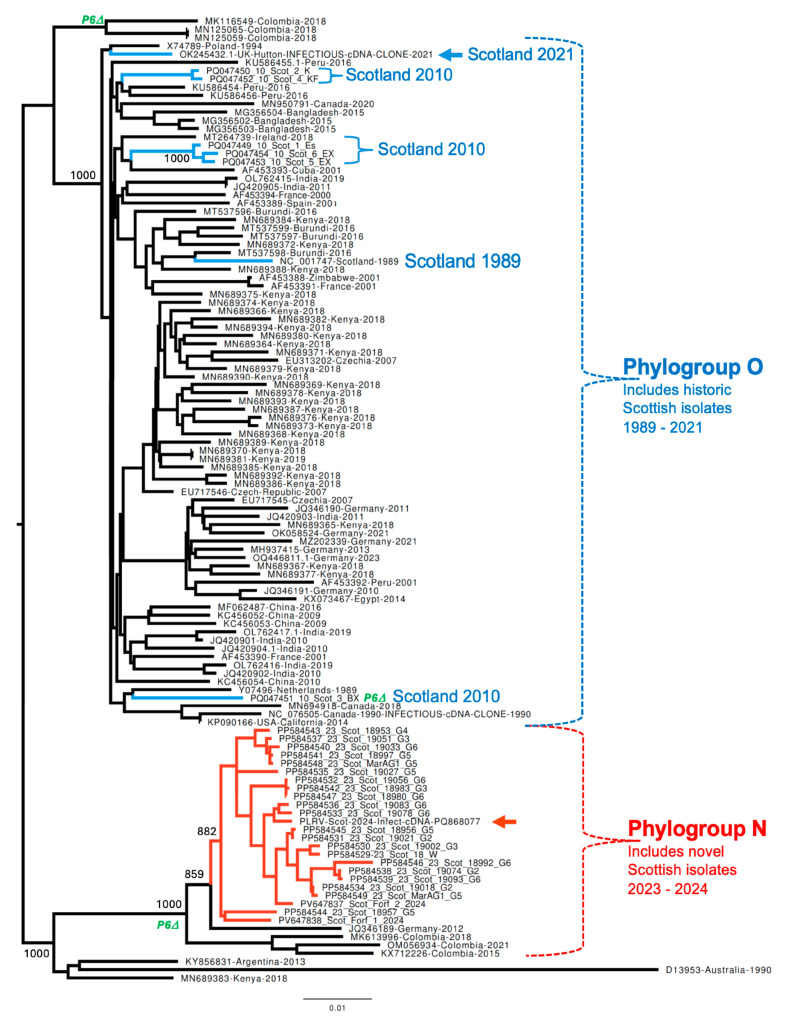
Sequence analysis of PLRV variants identified in Scotland and worldwide. Maximum likelihood (ML) phylogenetic tree for full-length nucleotide sequences of PLRV produced in this study (*n* = 30) and for all full-length PLRV genomes available in GenBank sampled globally (*n* = 87). Blue branches indicate Scottish PLRV samples collected from 1989 to 2021; red branches indicate all PLRV samples collected in Scotland during 2023 and 2024. Bootstrap values obtained after 1000 re-samplings are shown for selected nodes. PLRV variants with truncated P6 are indicated with green “P6D” at the node label or at the base of branch for the groups sharing this feature. The node labels include GenBank accession numbers, geographic location, and the year of sampling.

Phylogenetic analysis of the nucleotide (NT) sequences of PLRV identified in our study and all full-length PLRV sequences available in GenBank showed that all Scottish seed potato samples collected in the years 2023 and 2024 (Table 1) were infected with PLRV variants phylogenetically distinct from those collected in Scotland from 1989 to 2021, including PLRV variants originating from the 2010 samples that were sequenced in this study (Figure 1). The genomes of all Scottish PLRV sequences identified in 2023 and 2024 form a separate monophyletic clade with 89% bootstrap support, suggesting a monophyletic origin and perhaps recent diversification (Figure 1, red branches). These sequences were similar to the PLRV isolate identified in Germany in 2012 (GenBank accession JQ346189) [24]. The Scottish PLRV isolated in 2023 and 2024, the German 2012 variant, and three Columbian isolates identified in 2015–2021 clustered together to form a clade with 100% bootstrap support, which we hereafter refer to as PLRV phylogroup N (Figure 1).

All the full-length PLRV genomes of Scottish isolates sequenced from 1989 to 2021, including contigs from PLRV-infected plants originating from the 2010 field-collected plants which were tuber-propagated in an aphid-proof glasshouse until 2018–2023 (Figure 1, blue branches), belonged to a phylogenetic clade with 100% bootstrap support, which also included PLRV sequences from all potato-growing areas worldwide. The Scottish PLRV isolates from this clade, which we refer to as PLRV type O (Figure 1), also included a 2021 isolate for which an infectious cDNA clone was constructed [22]. We further tested the presence of both PLRV types in the RNA extracts isolated from Scottish potato samples field-collected in 2019, 2021, and 2022 by strain-specific RT-PCR amplification and sequencing of a 360 nt section of ORF5. This analysis showed that both PLRV phylogroups O and N were present in Scotland in 2019 (Supplementary Figure S1), suggesting gradual variant replacement.

**Figure 2:**
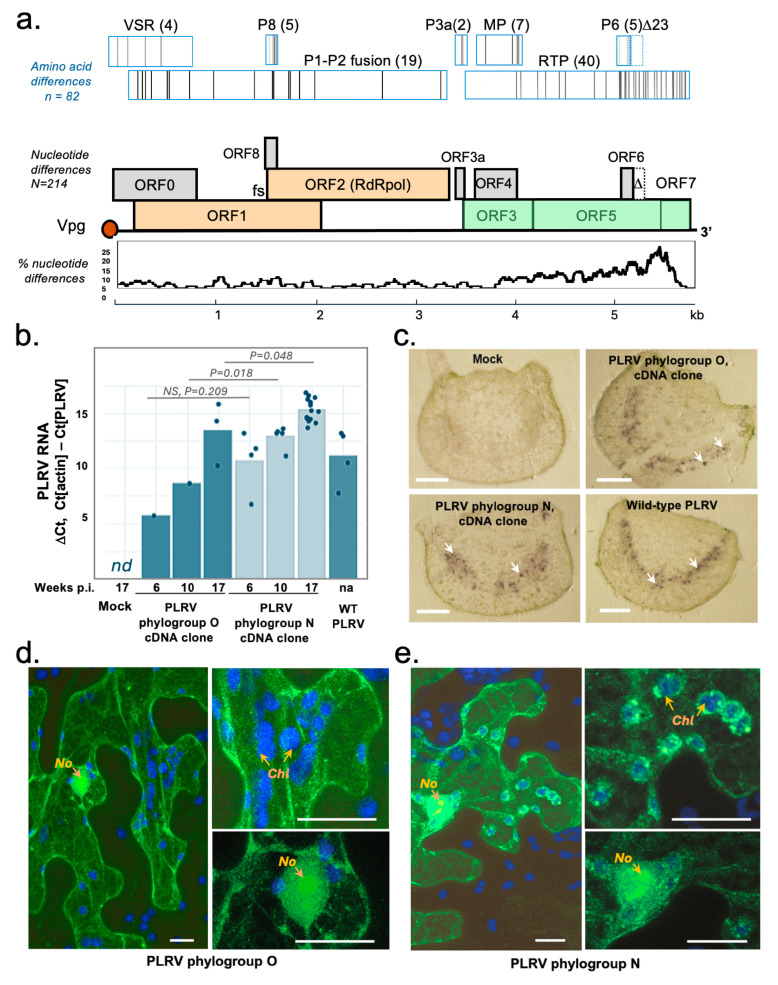
Comparative analysis of the novel and historic Scottish PLRV variants. (**a**) Organization of the PLRV genomic RNA and genetic divergence between infectious cDNA clones of the phylogroups O and N (GenBank accession numbers OK245432 and PQ868077, respectively). The graph below the PLRV genome map shows the percentage of nucleotide differences in a 100 nt sliding window. The differences between deduced AA sequences of the PLRV proteins are indicated as bars in the rectangles above the corresponding ORFs; numbers in parentheses show the number of differences in a given protein; the deletion of the C-terminal 23 AAs in P6 in the novel genotype N isolate is indicated with Δ, with the position of the P6 ORF in genotype O indicated by a dashed line. (**b**) Infection dynamics of the cDNA clone-derived PLRV of the phylogroups O and N in potato plants (cv Desiree). A plant agroinoculated with the empty binary vector was used as a negative control. The agroinfiltrated plants were sampled 6, 10, and 17 weeks postinfection (wpi). The levels of PLRV genomic RNA and potato actin mRNA transcript were quantified by RT-qPCR. ΔCt values for the PLRV RNA were normalized to the beta-actin mRNA. For the plants agroinfected with the PLRV clones, results are shown only for the plants which developed PLRV infection. A potato plant, cv 18WC, naturally infected with wild-type PLRV of the phylogroup N was used as a positive control. The statistical significance of the differences between PLRV levels in the plants agroinfected with the phylogroups O and N cDNA clones at the same timepoints is shown above the bars. Dots indicate individual DCt values; for 17 wpi, three samples were collected per experimental plant. (**c**) Immunoprints of potato petiole cross-sections probed with polyclonal antibodies to PLRV. White arrows indicate typical PLRV-infected phloem elements. Bars, 1 mm. (**d**,**e**) Intracellular localization of the GFP-tagged structural proteins of PLRV phylogroups O and N analyzed by confocal laser scanning microscopy. The GFP was fused to the N-termini of the ORF3 proteins; pseudocolors: green—GFP fluorescence, blue—chloroplast autofluorescence; No—nucleolus, Chl—chloroplast; bars, 10 mm.

Figure 2a exemplifies the genetic differences between PLRV phylogroups O and N by comparing the full-length infectious cDNA clones of PLRV type O (isolated in Scotland in 2021 [22]) and PLRV type N (isolated in Scotland in 2024). The sequences show overall 4% NT divergency, resulting in 82 divergent AAs in the putative proteins encoded by ORFs 0 to 8. This level of AA divergency (3.7%) is well below the 10% species demarcation threshold established by the ICTV. Therefore, the novel virus sequences should be classified as *Polerovirus PLRV,* rather than representing a new species. The highest sequence variation was observed in the 3′-parts of the ORF5 coding for the C-terminal, unstructured part of the readthrough protein (RTP), which is also expressed as the P7 protein. Several AA changes were found in the ORF0 protein (P0), which functions as a viral suppressor of RNA silencing (VSR) [5], and in the P1 protein, a part of RNA replicase (Figure 2a). Recently, it was experimentally demonstrated that the P0, P1, and P7 proteins of PLRV alter interaction between aphids and *Nicothiana benthamiana* plants by modulating production of phytohormones affecting aphid fecundity and attraction to PLRV-infected plants [25]. A noticeable feature of all PLRV isolates of the Scottish phylogroup N, which was also found in the German 2012 isolate and three South American isolates, was the stop codon in ORF6 shortening the P6 protein from 62 to 39 AAs. Such a truncated ORF6 was found only in 5 out of 89 full-length PLRV sequences outside of PLRV phylogroup N (Figure 1).

### 3.2. Infection Dynamics of cDNA Clone-Derived PLRV of Phylogroups O and N

We constructed a full-length infectious cDNA clone of PLRV type N using the PLRV RNA extracted from the symptomatic potato plants sourced in Scotland in 2024. The full-length genomic cDNA was cloned into the same binary vector backbone as the one used for the construction of the PLRV clone of the Scottish phylogroup O isolate in 2021 [22]. Replication dynamics of the cloned PLRV variants were assessed in the PLRV-susceptible potato plants, cv Desiree. To inoculate potato plants with the cDNA clone-derived PLRV, fresh potato stem cuttings were incubated in the agrobacterium cultures transformed with the viral cDNA construct and subsequently rooted to produce plants as described in [22]. The PLRV levels were quantified 6, 10, and 17 weeks after inoculation by RT-qPCR using the primers designed to detect both the historic and novel PLRV types (Figure 2b). Although agrobacterium suspensions for both PLRV cDNA clones used in agroinoculation had the same optical density, we observed a higher proportion of the plants which developed the virus infection for the phylogroup N PLRV construct (5 infected, 7 uninfected) compared with the O type PLRV clone (1 infected, 11 uninfected). The virus levels were significantly higher in the plants agroinfected with the construct of the phylogroup N compared to the phylogroup O (Figure 2b) at 10 and 17 weeks post-inoculation. Accumulation levels of both cDNA clone-derived PLRV variants in these plants (cv Desiree) were similar to or exceeded those observed in the potato cv 18WC infected with a wild-type PLRV (GenBank Accession PP584529).

Immunoprints of the petiole cross-sections probed with antisera to PLRV were used to assess the localization of the clone-derived and wild-type PLRV in potato plants agroinoculated 17 weeks previously. Both PLRV cDNA clone-derived types, as well as wild-type PLRV, were found in the phloem elements (Figure 2c). There were no visible differences in the number or distribution of the virus-infected phloem elements in the case of either cDNA clone-derived or wild-type PLRV (Figure 2c), while no PLRV was detected in the control, mock-inoculated potato plants.

### 3.3. Intracellular Localization of the Structural Proteins of PLRV of the Phylogroups O and N

The highest divergence between the proteins encoded by the O and PLRV types was observed for the RTP (40 of 82 divergent AAs), with most of them in the C-terminal part of the ORF5-encoded section. Information on the intracellular localization of proteins could provide clues on protein functions. We transiently expressed the CP and RTD (encoded by ORF3 and ORF5) blocks of both PLRV types with the green fluorescent protein (GFP) fused at the N-terminus of the CP in *N. benthamiana* leaves using an agrobacterium-mediated infiltration approach. The plasmid constructs for the expression of the GFP-tagged PLRV proteins contained the natural amber stop codon at the end of the PLRV ORF3, which is expected to result in expression of the GFP-tagged CP (encoded by ORF3) and the RTP (encoded by ORF3 and ORF5). Confocal laser scanning microscopy of the epidermal and mesophyll cells of the agroinfiltrated *N. benthamiana* leaves showed that the GFP-tagged proteins were localized to the nucleus and nuclei targeting nucleoli in the case of both PLRV phylogroups O and N (Figure 2d,e), similar to previously described localization of the GFP-tagged ORF3 protein of the Canadian PLRV isolate [26]. The major difference in intracellular localization between the novel and historic PLRV types was observed in the cytoplasm, where small GFP-tagged aggregates were associated with chloroplasts in the case of the novel PLRV type (Figure 2e), while there was no chloroplast association for the phylogroup O PLRV protein (Figure 2d). Differences in localization may indicate distinct biological properties of the structural proteins encoded by PLRV phylogroups N and O. Attachment to the chloroplast membrane was reported for many proteins of plant viruses [27,28]; however, the functional relevance of such interactions between chloroplasts and PLRV structural proteins remains to be elucidated.

### 3.4. Analysis of Selection Pressures Acting on the PLRV Genome

**Figure 3:**
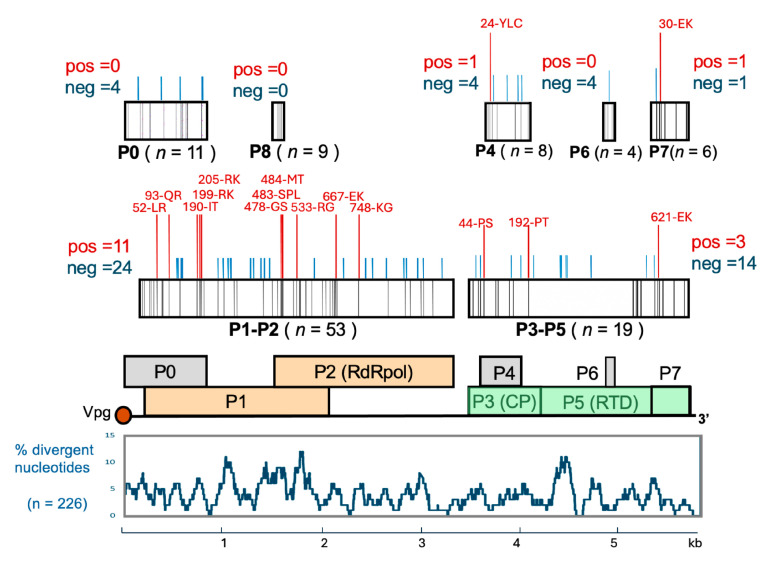
Positively and negatively selected sites in the proteins of the PLRV isolates from Scottish seed potato samples in 2023. Positively and negatively selected sites with posterior probability > 0.9 identified by FUBAR are shown as red or blue bars, respectively, above the boxes representing protein sequences, with amino acid changes and positions. Black bars within boxes representing protein sequences show divergent amino acid positions across all analyzed proteins. The total number of divergent amino acids (*n*) for a protein encoded by corresponding ORF is shown in bracketsThe percentage of divergent nucleotide (NT) positions for a 100 NT window is shown below the map of PLRV genomic RNA.

Phylogenetic analysis of the PLRV genomes from Scottish potato plants sampled in 2023 showed monophyletic origin and recent diversification after a bottleneck event. In total, across all 21 full-length contigs there were 3.8% divergent NT positions (*n* = 226), and 91 divergent AA positions in the putative P0, P1–P2, P3–P5 (RTP), P4, and P6 proteins (Figure 3). Using the FUBAR tool [20], we called 51 AA sites subjected to negative selection and 16 sites subjected to positive selection. The results of this analysis are summarized in Figure 2, which shows such sites with posterior probability exceeding 0.9. While the sites subjected to negative, stabilizing selection were distributed evenly across the genome, the highest number of positively selected sites (*n* = 8) was found in the ORF1-encoded part of the P1–P2 fusion protein. These were no positively selected sites in the ORF0-encoded protein, which acts as a suppressor of RNAi and interacts with the host proteins. We found three positively selected sites in the RTD protein encoded by ORF3 and ORF5. At one of these, AA position 621 in the P3–P5 fusion proteins, which corresponds to position 30 in the P7 protein, there was either lysine (K) or glutamate (E). We observed glutamate (E) at this position in higher proportion in the early field generation samples, FGs 2,3 (E/K–6/1), compared to the later field generations, FGs 4,5,6 (E/K–10/4), using the Fisher exact probability test, two-tailed, *p* = 0.0237. The C-terminal part of the PLRV RTD could be involved in interaction with components of phloem and aphids; therefore, aphid transmission and phloem movement may favor E621 or K621, respectively. The high variability of P1 and RTD in PLRV phylogroup N observed in our study (Figure 3) was in good agreement with the results of the global PLRV diversity analysis, which also identified RTD and P1 as the proteins with the highest percentages of mutations [5].

### 3.5. Possible Impact of Transmission Routes on PLRV Diversification

**Figure 4:**
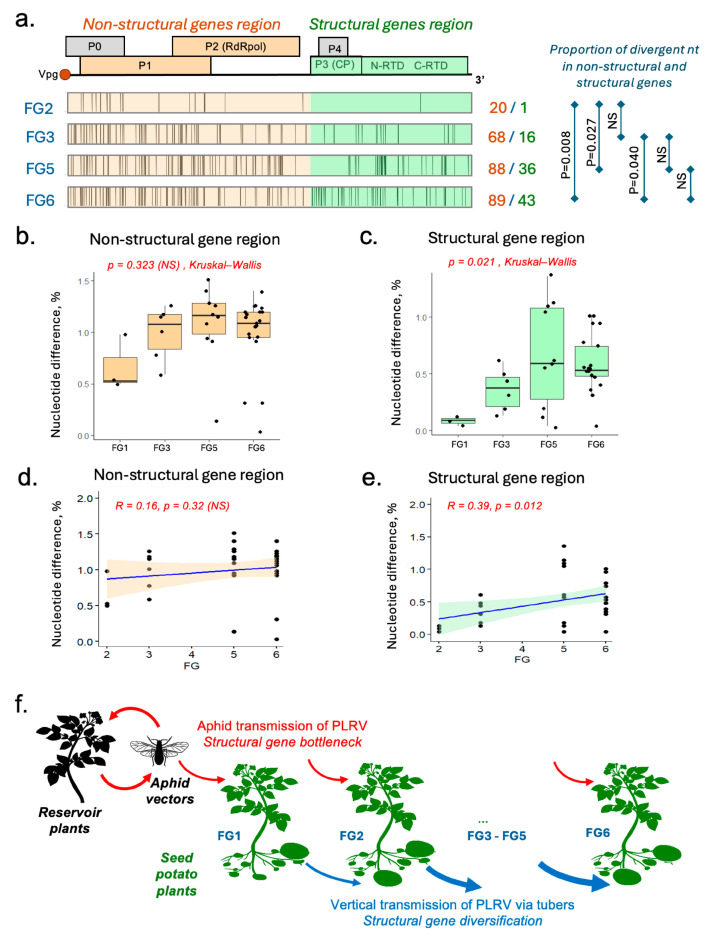
Analysis of selection pressures on the PLRV genome imposed by the aphid and tuber transmission routes. (**a**) Distribution of divergent nucleotide (NT) positions, represented as vertical bars, in the PLRV genomes within the same FG for the de novo contigs. The schematic representation of the PLRV genome is shown above. The numbers of divergent NTs in the genome sections for FGs (non-structural section/structural section) and the significance of the differences in the divergent NT proportions in the non-structural and structural sections, assessed by contingency table analysis, are shown on the right side. (**b**–**e**) Analysis of PLRV divergence within FGs for the de novo contigs. The boxplot shows pairwise NT divergence, calculated for the sections of the PLRV genome: (**b**) non-structural gene region (1–3.5 kb region), (**c**) structural gene region (3.5–5.9 kb region). Dots represent the values for individual pairwise comparisons between libraries within each of the FGs. (**d**,**e**) Scatter plots for the pairwise NT divergence values within an FG. Shown are the Pearson correlation coefficient (R) and significance level. (**f**) Proposed model of PLRV circulation. Plants of the first field generation (FG1) are grown from virus-free tubers; the progeny tubers are planted next season to produce FG2. The cycle continues up to FG6. Early FGs are infected by aphids; later FGs could be infected with PLRV received through tubers from infected parental plants. Red arrows indicate aphid transmission; blue arrows indicate vertical transmission via tubers, with arrow thickness reflecting the relative contribution of tuber-mediated transmission. Figure 4f is adapted from [29].

Analysis of de novo contigs produced for the libraries showed variation in the divergency levels in the PLRV genome regions containing the structural and non-structural genes within FGs (Figure 4a). We observed that in early FGs the ratio between the numbers of divergent NTs in the non-structural genes section and in the structural genes section (20 to 1 in FG2, 68 to 16 for FG3) was higher than in the late FGs (88 to 36 for FG5, 89 to 43 for FG6) (Figure 4a). Contingency table analysis showed that these differences were statistically significant between early and late FGs. For FG6, the proportion of the divergent positions in the structural genes section was significantly higher compared to those in FG2 and FG3 (*p* = 0.008 and *p* = 0.040, correspondingly). A significant difference in relative distribution of divergent NTs was also observed between FG2 and FG5 (*p* = 0.027) (Figure 4b).

We calculated pairwise percentages of nucleotide divergence between de novo contigs within FGs 2, 3, 5, and 6 separately for the PLRV genome sections containing non-structural genes (positions 1–3.5 kb) (Figure 4c,d). We observed significant differences in the structural gene section in the late FGs (Kruskal–Wallis test, c^2^ = 9.7271, df = 3, *p*-value = 0.0210), while the percentages of nucleotide divergence for the non-structural gene section (5′ proximal section part of the genomic RNA, 1–35 kb) were not significantly different across all FGs (Kruskal–Wallis test, c^2^ = 4.3999, df = 3, *p* = 0.2214). Our analysis showed a significant increase in diversity of the structural gene block in the later FGs (*p* < 0.05), while diversity of the non-structural genes did not show a significant change across FGs (Figure 4e,f).

Based on these results we suggest a hypothetical model of PLRV transmission in seed potatoes and the impact of transmission routes on the genetic diversity of PLRV (Figure 4f). Potato plants could be infected with PLRV by aphids or could acquire PLRV via tuber transmission from infected mother plants. FG1 plants are infected only by aphids, but plants of later FGs could be infected either by aphid vectors (horizontal transmission), or through tuber propagation (vertical transmission). Reduced diversity of the structural genes in the early FG2 and FG3, which are more likely to receive the virus from aphids rather than from tubers compared to the later FG5 and FG6, may suggest that aphid transmission imposes a genetic bottleneck acting on the genes of structural proteins involved in interaction with aphids.

## 4. Discussion

Epidemics of vector-borne plant viruses can have a significant economic impact by affecting yield and quality of crops, but their causes are not fully understood [14]. Several factors could contribute to the increase of virus incidence, of which introduction and emergence of novel virus variants and changes in insect vector populations are the most important drivers [30].

In this study we analyzed the genetic diversity of PLRV circulating in Scottish potato crops. High-throughput sequencing of PLRV genomes from potato samples sourced in the years 2023 and 2024 showed that the phylogroup N of PLRV has displaced phylogroup PLRV isolates of phylogroup O, which was prevalent in Scottish potatoes since at least 1989 (Figure 1) [2,22]. All Scottish PLRV isolates sourced in 2023–2024 showed the highest similarity with the PLRV isolate identified in Germany in 2012, which was reported as causing asymptomatic infection in some potato cultivars in glasshouse conditions [24]. Although all Scottish 2023–2024 PLRV isolates of the phylogroup N sequenced in this study were identified in field-grown symptomatic potato plants, there is a possibility that different pathogenic characteristics of the phylogroup N and/or other environmental or cultural factors might have contributed to selection and prevalence of the phylogroup N [31].

Sequence analysis showed that the RNA genomes of Scottish PLRV isolates of the phylogroups O and N have approximately 4% divergent NTs (Figure 2a). Comparison of the proteins encoded by the infectious cDNA clones of these types (Figure 1, pointed out by arrows) showed 82 divergent AA positions in all proteins, with the largest number located in the C-terminal part of the RTP and P7 (Figure 2a). Another feature of the isolates belonging to PLRV phylogroup N is a truncation of P6 due to NT substitution leading to introduction of a stop codon resulting in the deletion of 23 C-terminal AAs of the originally 62 AA long protein.

These AA differences may lead to the alteration of the biological properties of the virus isolated. For example, the RTP is a minor component of the PLRV capsid which interacts with both plant and aphid components [5,9]. Therefore, changes in the RTP may affect virus movement within plants and possibly aphid transmission. Indeed, comparison of infection dynamics of the cDNA clone-derived PLRV in potato plants suggested that the PLRV clone of the phylogroup N replicated to a higher level compared to the phylogroup O (Figure 2b). Further studies in natural conditions of transmission and plant growth are needed to confirm these preliminary observations. Also, localization studies showed that structural proteins of the phylogroups N and O PLRV have some differences in intracellular localization (Figure 2d,e). The P0, P1, and P7 proteins of PLRV are involved in changing attraction of aphids to infected plants by affecting aphids’ fecundity via modulation of phytohormone production in *N. benthamiana* plants [25]; therefore, differences between PLRV phylogroups O and N may have an effect on the virus’s ability to be transmitted by the aphid vectors.

Phylogenetic analysis of PLRV from infected seed potatoes sampled in 2023 showed monophyletic origin (Figure 1), making it possible to analyze selective pressure action on the PLRV genomes. In total, 51 AA sites were subjected to negative selection and 16 sites were subjected to positive selection in the P0, P1-P2 fusion, P3-P5 (RTP), P4, and P6 proteins (Figure 3). We found no positively selected sites in the P0 protein, which acts as a suppressor of antiviral RNAi. The action of P0 involves interaction with the host S-phase kinase-related protein-1 which is required to direct ubiquitination and degradation of Argonaute1 [32]. Such interaction requires adaptation to a specific plant host; therefore, the lack of diversification of P0 may indicate that circulation of PLRV involves solely potato plants. Positively selected sites in P3 and the RTP, as well as in P7, such as position 621 in the RTP (Figure 3), may reflect adaptation of PLRV to phloem transmission following the aphid transmission step.

PLRV is transmitted from virus-infected to virus-free plants by aphids, principally by *Myzus persicae,* which vector the virus in a non-replicative circulative manner [4,5]. Tuber propagation of potatoes also allows transmission of PLRV from infected plants to the next vegetative generations [33]. Seed potato production starts with planting virus-free mini-tubers, from which the progeny tubers are subsequently propagated over several FGs. Therefore, all PLRV-infected plants of FG1 are infected only via aphid transmission, while the later FGs either could be infected by the aphids or could have acquired PLRV vertically from parental infected plants via infected tubers (Figure 4f). In this study we investigated the impacts of vertical (tuber) and horizontal (aphid) transmission on the genetic diversity of PLRV via high-throughput sequencing of PLRV from different FGs of seed potatoes. Plants of early FGs (FG2 and FG3) were more likely to be directly infected with PLRV by the aphid vectors, while the late FGs (FG4 to FG6) were likely to be infected with the virus propagated via tuber transmission, which underwent up to five field generations.

We found that recently aphid-transmitted PLRV variants from FG2 and FG3 had significantly lower genetic diversity of 3′ genome section coding for the structural proteins compared to those from the late field generations, while diversity levels of the non-structural gene block were not significantly different across all FGs, from FG2 to FG6 (Figure 4a–e). This suggests that aphid transmission may impose a strong genetic bottleneck on the structural genes of PLRV, leading to the reduction in diversity. This could be driven by selection of the PLRV variants with structural genes better adapted to aphid transmission. The structural genes showed diversification in the later FGs, which might be a result of the absence of the need to be adapted to aphid transmission and/or possible adaptation to tuber transmission (Figure 4a–e). Notably, the positively selected divergent aa 621 in the RTP which showed significant association with field generation (glutamate in FG2, 3, and lysine in FG4,5,6) is located in the region of RTD, mutation in which affects aphid transmission, possibly by affecting passage through the aphid gut membrane [34,35]. We hypothesized that transmission of PLRV via tubers for several vegetative generations (FGs) in the absence of the aphid transmission events may result in accumulation of the genetic variants of PLRV with decreased adaptation to aphid vectoring and/or higher adaptation to vertical transmission (Figure 4f).

The lack of a genetic bottleneck associated with aphid transmission which acted on the non-structural genes (such as ORF0, 1, and 2) was in agreement with the non-replicative nature of the aphid transmission of the virus and the lack of interaction of the replicative genes of PLRV with the aphid components. The impact of such diversification of PLRV on the virus’s pathogenicity and ability to be transmitted by aphid vectors, and possible implications for the virus’s epidemiology, requires further investigation.

## 5. Conclusions

We identified a prevalent PLRV phylogroup in Scottish potato crops which, by 2023–2024, had displaced a different phylogroup present for over four decades. The currently prevalent PLRV type is phylogenetically related to a German isolate identified in 2012. Analysis of PLRV populations from different field generations of seed potatoes showed a reduction in genetic diversity of the structural genes in PLRV recently transmitted by aphids, which suggests a strong genetic bottleneck acting on the structural genes of PLRV.

## Figures and Tables

**Table 1:** PLRV samples used in this study.

Sample ID	Field Sampling Year	Region of Scotland	Location	Field Generation	GenBank Accession Number ^1^
2023_18_W	2023	East Central	Forfar	FG6	PP584529
2023_19002	2023	Northeast	Inverurie	FG3	PP584530
2023_19021	2023	Northeast	Inverurie	FG2	PP584531
2023_19056	2023	East Central	Perth	FG6	PP584532
2023_19078	2023	Northeast	Inverurie	FG6	PP584533
2023_19018	2023	Northeast	Inverurie	FG2	PP584534
2023_19027	2023	Southeast	Galashiels	FG5	PP584535
2023_19083	2023	Northeast	Inverurie	FG6	PP584536
2023_19051	2023	East Central	Perth	FG3	PP584537
2023_19074	2023	Northeast	Inverurie	FG2	PP584538
2023_19093	2023	East Central	Perth	FG6	PP584539
2023_19033	2023	East Central	Perth	FG6	PP584540
2023_18997	2023	Northeast	Inverurie	FG5	PP584541
2023_18983	2023	Northeast	Inverurie	FG3	PP584542
2023_18953	2023	East Central	Perth	FG4	PP584543
2023_18957	2023	East Central	Perth	FG5	PP584544
2023_18956	2023	East Central	Perth	FG5	PP584545
2023_18992	2023	Northeast	Inverurie	FG6	PP584546
2023_18980	2023	East Central	Perth	FG6	PP584547
2023_MarAG1	2023	East Central	Fife	FG5	PP584548
2023_MarAG2	2023	Northeast	Aberdeen	FG3	PP584549
2010_1_Es	2010 ^2^	East Central	Gogarbank	Ware	PQ047449
2010_2_K	2010 ^2^	East Central	Gogarbank	Ware	PQ047450
2010_3_BX	2012 ^2^	East Central	Gogarbank	Ware	PQ047451
20104_KF	2010 ^2^	East Central	Gogarbank	Ware	PQ047452
20105_EX	2010 ^2^	East Central	Gogarbank	Ware	PQ047453
2010_EX	2010 ^2^	East Central	Gogarbank	Ware	PQ047454
2024_cDNA_clone	2024	East Central	Forfar	Ware	PQ868077 *
2024_Forfar_1	2024	East Central	Forfar	Ware	PV647838 **
2024_Forfar_2	2024	East Central	Forfar	Ware	PV647837 **

^1^ GeneBank accession numbers of de novo assembled contigs, cloned cDNA (marked with *), or RT-PCR amplicons (marked with **); ^2^ RNA from the potato plants propagated in an insect-proof greenhouse since 2010 was extracted in 2019 (for 2010_1_Es), 2018 (for 2010_2_K), and 2023 (for the rest of the samples).

## Data Availability

The nucleotide sequences of PLRV isolates produced in this study were deposited in GenBank, accession numbers PP584529-PP584549, PQ047449-PQ047454, PV647837, and PV647838.

## Supplementary Materials

The following supporting information can be downloaded at https://www.mdpi.com/article/10.3390/v17101294/s1: Figure S1: Identification of PLRV of the phylogroups O and N in the Scottish potato samples collected in 2019–2022.
