# Supplementary material for: Genetic Diversity of Potato Leafroll Virus (Polerovirus PLRV) Is Shaped by Variant Displacements and Selective Pressures Imposed by Aphid and Tuber Transmission Routes
Source: Viruses. 2025 Sep 24;17(10):1294. doi: 10.3390/v17101294 (PMC12568242; doi:10.3390/v17101294)
Supplement: Supplementary file 1 [file viruses-17-01294-s001.zip › viruses-3864071-supplementary.pdf]

Supplementary Figure S1. Identification of PLRV of the phylogroups O and N in the Scottish potato samples collected in 2019 - 2022.

A. Phylogeny and alignment of the partial nucleotide sequences of the 3' sections of the Scottish isolates, year of collection indicated by prefix in the sequence ID; also included the corresponding regions of the PLRV type O (GenBank Accession OK245432), and the PLRV type N (GenBank Accession PQ868077).

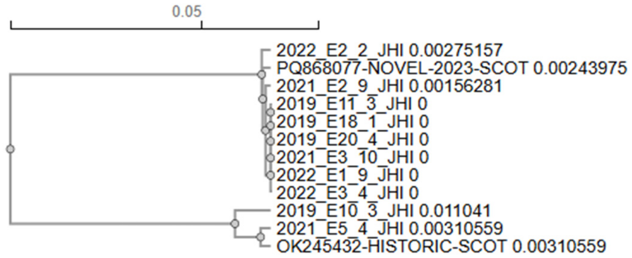


```
2022_E2_2_JHI                 ---------------------------------------TTTTAGAAAATTGGGATGCTG          21
PQ868077-NOVEL-2023-SCOT      ATTCGAGAACTGTTACTGACTTTTCTCCGAAAGCCGATCTTTTAGAAAATTGGGATGCTG          5279
2021_E2_9_JHI                 ---------------------------------------TTTTAGAAAATTGGGATGCTG          21
2019_E11_3_JHI                ---------------------------------------TTTTAGAAAATTGGGATGCTG          21
2019_E18_1_JHI                ---------------------------------------TTTTAGAAAATTGGGATGCTG          21
2019_E20_4_JHI                ---------------------------------------TTTTAGAAAATTGGGATGCTG          21
2021_E3_10_JHI                ---------------------------------------TTTTAGAAAATTGGGATGCTG          21
2022_E1_9_JHI                 ---------------------------------------TTTTAGAAAATTGGGATGCTG          21
2022_E3_4_JHI                 ---------------------------------------TTTTAGAAAATTGGGATGCTG          21
2019_E10_3_JHI                ------------------------------------------TGGAGAATTGGGATGCCG          18
2021_E5_4_JHI                 ---------------------------------------TACTGGAGAATTGGGATGCCG          21
OK245432-HISTORIC-SCOT        ATTCAAGAACTGTTACTGACTTTTCTCCGAAAGCCGATCTACTGGAGAATTGGGATGCCG          5280
                                                                        * ** *********** *

2022_E2_2_JHI                 AATACTTCGACCCTGGTTATTCAAAGGAAGACGTCGCAGCTGCTACTCTTATAGCACACG          81
PQ868077-NOVEL-2023-SCOT      AATACTTCGACCCTGGTTATTCAAAGGAAGACGTCGCAGCTGCTACTCTTATAGCACACG          5339
2021_E2_9_JHI                 AATACTTCGACCCTGGTTATTCAAAGGAAGACGTCGCAGCTGCTACTCTTATAGCACACG          81
2019_E11_3_JHI                AATACTTCGACCCTGGTTATTCAAAGGAAGACGTCGCAGCTGCTACTCTTATAGCACACG          81
2019_E18_1_JHI                AATACTTCGACCCTGGTTATTCAAAGGAAGACGTCGCAGCTGCTACTCTTATAGCACACG          81
2019_E20_4_JHI                AATACTTCGACCCTGGTTATTCAAAGGAAGACGTCGCAGCTGCTACTCTTATAGCACACG          81
2021_E3_10_JHI                AATACTTCGACCCTGGTTATTCAAAGGAAGACGTCGCAGCTGCTACTCTTATAGCACACG          81
2022_E1_9_JHI                 AATACTTCGACCCTGGTTATTCAAAGGAAGACGTCGCAGCTGCTACTCTTATAGCACACG          81
2022_E3_4_JHI                 AATACTTCGACCCTGGTTATTCAAAGGAAGACGTCGCAGCTGCTACTCTTATAGCACACG          81
2019_E10_3_JHI                AACACTTTGACCCTGGTTATTCCAAAGAAGATGTCGCTGCCGCTACTATTATAGCGCACG          78
2021_E5_4_JHI                 AACACTTCGACCCTGGTTATTCCAAAGAAGATGTCGCTGCTGCTACTATTATAGCACACG          81
OK245432-HISTORIC-SCOT        AACACTTCGACCCTGGTTATTCCAAAGAAGATGTCGCTGCTGCTACTATTATAGCGCACG          5340
                              ** **** ************** ** ***** ***** ** ****** ******* ****

2022_E2_2_JHI                 GCAGTATTCAGGATGGGCGAAGCATGTTGGAGAAGAGAGAGGAAAGTGTCAAACGCAAAA          141
PQ868077-NOVEL-2023-SCOT      GCAGTATTCAGGATGGGCGAAGTATGTTGGAGAAGAGAGAGGAAAGTGTCAAACGCAAAA          5399
2021_E2_9_JHI                 GCAGTATTCAGGATGGGCGAAGTATGTTGGAGAAGAGAGAGGAAAGTGTTAAACGCAAAA          141
2019_E11_3_JHI                GCAGTATTCAGGATGGGCGAAGTATGTTGGAGAAGAGAGAGGAAAGTGTCAAACGCAAAA          141
2019_E18_1_JHI                GCAGTATTCAGGATGGGCGAAGTATGTTGGAGAAGAGAGAGGAAAGTGTCAAACGCAAAA          141
2019_E20_4_JHI                GCAGTATTCAGGATGGGCGAAGTATGTTGGAGAAGAGAGAGGAAAGTGTCAAACGCAAAA          141
2021_E3_10_JHI                GCAGTATTCAGGATGGGCGAAGTATGTTGGAGAAGAGAGAGGAAAGTGTCAAACGCAAAA          141
2022_E1_9_JHI                 GCAGTATTCAGGATGGGCGAAGTATGTTGGAGAAGAGAGAGGAAAGTGTCAAACGCAAAA          141
2022_E3_4_JHI                 GCAGTATTCAGGATGGGCGAAGTATGTTGGAGAAGAGAGAGGAAAGTGTCAAACGCAAAA          141
2019_E10_3_JHI                GCAGTATTCAAGATGGGCGAAGTATGTTGGAGAAGAGAGAGGAAAATGTCAAGAACAAAA          138
2021_E5_4_JHI                 GCAGTATTCAAGATGGGCGAAGTATGTTGGAGAAGAGAGAGGAAAATGTCAAGAACAAAA          141
OK245432-HISTORIC-SCOT        GCAGTATTCAAGATGGGCGAAGTATGTTGGAGAAGAGAGAGGAAAATGTCAAGAACAAAA          5400
                              ********** *********** ********************** *** **   *****

2022_E2_2_JHI                 CCTCCTCCTGGAAGCCCCCGCTGCCTAAAGCGGTGAGCCCAGCCATAGCCGAATTGCGGT          201
PQ868077-NOVEL-2023-SCOT      CCTCCTCCTGGAAGCCCCCGCTGCCTAAAGCGGTGAGCCCAGCCATAGCCGAATTGCGGT          5459
2021_E2_9_JHI                 CCTCCTCCTGGAAGCCCCCGCTGCCTAAAGCGGTGAGCCCAGCCATAGCCGAATTGCGGT          201
2019_E11_3_JHI                CCTCCTCCTGGAAGCCCCCGCTGCCTAAAGCGGTGAGCCCAGCCATAGCCGAATTGCGGT          201
2019_E18_1_JHI                CCTCCTCCTGGAAGCCCCCGCTGCCTAAAGCGGTGAGCCCAGCCATAGCCGAATTGCGGT          201
2019_E20_4_JHI                CCTCCTCCTGGAAGCCCCCGCTGCCTAAAGCGGTGAGCCCAGCCATAGCCGAATTGCGGT          201
2021_E3_10_JHI                CCTCCTCCTGGAAGCCCCCGCTGCCTAAAGCGGTGAGCCCAGCCATAGCCGAATTGCGGT          201
2022_E1_9_JHI                 CCTCCTCCTGGAAGCCCCCGCTGCCTAAAGCGGTGAGCCCAGCCATAGCCGAATTGCGGT          201
2022_E3_4_JHI                 CCTCCTCCTGGAAGCCCCCGCTGCCTAAAGCGGTGAGCCCAGCCATAGCCGAATTGCGGT          201
2019_E10_3_JHI                CTTCCTCCTGGAAGTCCCCGCTACCTAAAGCGGTGAGCCCAGCCATAGCCAAATTGCGCT          198
2021_E5_4_JHI                 CCTCCTCCTGGAAGCCCCCGTTACCTAAAGCGGTGAGCCCAGCCATAGCCAAATTGCGCT          201
OK245432-HISTORIC-SCOT        CCTCCTCCTGGAAGCCCCCGTTATCTAAAGCGGTGAGCCCAGCCATAGCCAAATTGCGCT          5460
                              * ************ ***** *  ************************** ******* *

2022_E2_2_JHI                 CAATCCGTAAGACCCAACCCCTCGAGGGTGGGACACTGAATAAGGGCACTACTGATGGTG          261
PQ868077-NOVEL-2023-SCOT      CAATCCGTAAGACTCAACCCCTCGAGGGTGGGACACTGAATAAGGGCACTACTGATGGTG          5519
2021_E2_9_JHI                 CAATCCGTAAGACCCAACCCCTCGAGGGTGGGACACTGAATAAGGGCACTACTGATGGTG          261
2019_E11_3_JHI                CAATCCGTAAGACCCAACCCCTCGAGGGTGGGACACTGAATAAGGGCACTACTGATGGTG          261
2019_E18_1_JHI                CAATCCGTAAGACCCAACCCCTCGAGGGTGGGACACTGAATAAGGGCACTACTGATGGTG          261
2019_E20_4_JHI                CAATCCGTAAGACCCAACCCCTCGAGGGTGGGACACTGAATAAGGGCACTACTGATGGTG          261
2021_E3_10_JHI                CAATCCGTAAGACCCAACCCCTCGAGGGTGGGACACTGAATAAGGGCACTACTGATGGTG          261
2022_E1_9_JHI                 CAATCCGTAAGACCCAACCCCTCGAGGGTGGGACACTGAATAAGGGCACTACTGATGGTG          261
2022_E3_4_JHI                 CAATCCGTAAGACCCAACCCCTCGAGGGTGGGACACTGAATAAGGGCACTACTGATGGTG          261
2019_E10_3_JHI                CGATTCGCAAATCCCAACCCCTCGAGGGAGGGACCCTTAAGAAAGACGCCACTGATGGTG          258
2021_E5_4_JHI                 CGATTCGCAAATCCCAACCCCTCGAGGGAGGGACCCTTAAGAAAGACGCTACTGATGGTG          261
OK245432-HISTORIC-SCOT        CGATTCGCAAATCCCAACCCCTCGAGGGAGGGACCCTTAAGAAAGACGCTACTGATGGTG          5520
                              * ** ** **  * ************** ***** ** ** ** * * * **********
```

```
2022_E2_2_JHI                 CCTCAACAATTGGCAGTGGTTCTCTAACTGGTGGTACGCTCAAAAGGAAACAGACTT---     318
PQ868077-NOVEL-2023-SCOT      CCTCAACAATTGGCAGTGGTTCTCTAACTGGTGGTACGCTCAAAAGGAAACAGACTTATG     5579
2021_E2_9_JHI                 CCTCAACAATTGGCAGTGGTTCTCTAACTGGTGGTACGCTCAAAAGGAAACAGACTTAT-     320
2019_E11_3_JHI                CCTCAACAATTGGCAGTGGTTCTCTAACTGGTGGTACGCTCAAAAGGAAACAGACTT---     318
2019_E18_1_JHI                CCTCAACAATTGGCAGTGGTTCTCTAACTGGTGGTACGCTCAAAAGGAAACAGACTTAT-     320
2019_E20_4_JHI                CCTCAACAATTGGCAGTGGTTCTCTAACTGGTGGTACGCTCAAAAGGAAACAGACTTAT-     320
2021_E3_10_JHI                CCTCAACAATTGGCAGTGGTTCTCTAACTGGTGGTACGCTCAAAAGGAAACAGACTTATG     321
2022_E1_9_JHI                 CCTCAACAATTGGCAGTGGTTCTCTAACTGGTGGTACGCTCAAAAGGAAACAGACTTAT-     320
2022_E3_4_JHI                 CCTCAACAATTGGCAGTGGTTCTCTAACTGGTGGTACGCTCAAAAGGAAACAGACTTAT-     320
2019_E10_3_JHI                TCTCATCTATTGGCAGTGGTTCTCTAACAGGTGGCACGCTTAAGAGGAAGGTAACTGTT-     317
2021_E5_4_JHI                 TCTCATCTATTGGCAGTGGTTCTCTAACAGGTGGCACGCTTAAGAGGAAGGTAACTGTTG     321
OK245432-HISTORIC-SCOT        TCTCATCTATTGGCAGTGGTTCTCTAACAGGTGGCACGCTTAAGAGGAAGGTAACTGTTG     5580
                               **** * ******************* ***** ***** ** *****    ***

2022_E2_2_JHI                 ------------------------------------------------------------     318
PQ868077-NOVEL-2023-SCOT      AAGAGCGCTTGCTACAGACCTTAACAACTGAACAAAGGCTGTGGTATGAGAATTTAAAGA     5639
2021_E2_9_JHI                 ------------------------------------------------------------     320
2019_E11_3_JHI                ------------------------------------------------------------     318
2019_E18_1_JHI                ------------------------------------------------------------     320
2019_E20_4_JHI                ------------------------------------------------------------     320
2021_E3_10_JHI                AG----------------------------------------------------------     323
2022_E1_9_JHI                 ------------------------------------------------------------     320
2022_E3_4_JHI                 ------------------------------------------------------------     320
2019_E10_3_JHI                ------------------------------------------------------------     317
2021_E5_4_JHI                 A-----------------------------------------------------------     322
OK245432-HISTORIC-SCOT        AAGAGCGTTTACTGCAGACCTTAACAACTGAACAAAGGCTGTGGTACGAGAATTTGAAGA     5640
```

B. Phylogeny and alignment of the partial ORF5 protein sequences encoded by the 2019-2022 Scottish PLRV isolates.

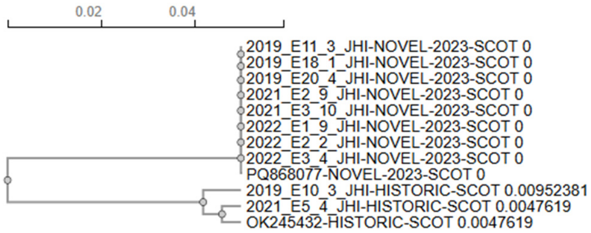


```
2019_E11_3_JHI-NOVEL-2023-SCOT      ------------------LENWDAEYFDPGYSKEDVAAATLIAHGSIQDGRSMLEKREES      42
2019_E18_1_JHI-NOVEL-2023-SCOT      ------------------LENWDAEYFDPGYSKEDVAAATLIAHGSIQDGRSMLEKREES      42
2019_E20_4_JHI-NOVEL-2023-SCOT      ------------------LENWDAEYFDPGYSKEDVAAATLIAHGSIQDGRSMLEKREES      42
2021_E2_9_JHI-NOVEL-2023-SCOT       ------------------LENWDAEYFDPGYSKEDVAAATLIAHGSIQDGRSMLEKREES      42
2021_E3_10_JHI-NOVEL-2023-SCOT      ------------------LENWDAEYFDPGYSKEDVAAATLIAHGSIQDGRSMLEKREES      42
2022_E1_9_JHI-NOVEL-2023-SCOT       ------------------LENWDAEYFDPGYSKEDVAAATLIAHGSIQDGRSMLEKREES      42
2022_E2_2_JHI-NOVEL-2023-SCOT       ------------------LENWDAEYFDPGYSKEDVAAATLIAHGSIQDGRSMLEKREES      42
2022_E3_4_JHI-NOVEL-2023-SCOT       ------------------LENWDAEYFDPGYSKEDVAAATLIAHGSIQDGRSMLEKREES      42
PQ868077-NOVEL-2023-SCOT            RVSGNSRTVTDFSPKADLLENWDAEYFDPGYSKEDVAAATLIAHGSIQDGRSMLEKREES      600
2019_E10_3_JHI-HISTORIC-SCOT        -------------------ENWDAEHFDPGYSKEDVAAATIIAHGSIQDGRSMLEKREEN      41
2021_E5_4_JHI-HISTORIC-SCOT         ------------------LENWDAEHFDPGYSKEDVAAATIIAHGSIQDGRSMLEKREEN      42
OK245432-HISTORIC-SCOT              RVSGDSRTVTDFSPKADLLENWDAEHFDPGYSKEDVAAATIIAHGSIQDGRSMLEKREEN      600
                                                       ******:**************:******************.

2019_E11_3_JHI-NOVEL-2023-SCOT      VKRKTSSWKPPLPKAVSPAIAELRSIRKTQPLEGGTLNKGTTDGASTIGSGSLTGGTLKR      102
2019_E18_1_JHI-NOVEL-2023-SCOT      VKRKTSSWKPPLPKAVSPAIAELRSIRKTQPLEGGTLNKGTTDGASTIGSGSLTGGTLKR      102
2019_E20_4_JHI-NOVEL-2023-SCOT      VKRKTSSWKPPLPKAVSPAIAELRSIRKTQPLEGGTLNKGTTDGASTIGSGSLTGGTLKR      102
2021_E2_9_JHI-NOVEL-2023-SCOT       VKRKTSSWKPPLPKAVSPAIAELRSIRKTQPLEGGTLNKGTTDGASTIGSGSLTGGTLKR      102
2021_E3_10_JHI-NOVEL-2023-SCOT      VKRKTSSWKPPLPKAVSPAIAELRSIRKTQPLEGGTLNKGTTDGASTIGSGSLTGGTLKR      102
2022_E1_9_JHI-NOVEL-2023-SCOT       VKRKTSSWKPPLPKAVSPAIAELRSIRKTQPLEGGTLNKGTTDGASTIGSGSLTGGTLKR      102
2022_E2_2_JHI-NOVEL-2023-SCOT       VKRKTSSWKPPLPKAVSPAIAELRSIRKTQPLEGGTLNKGTTDGASTIGSGSLTGGTLKR      102
2022_E3_4_JHI-NOVEL-2023-SCOT       VKRKTSSWKPPLPKAVSPAIAELRSIRKTQPLEGGTLNKGTTDGASTIGSGSLTGGTLKR      102
PQ868077-NOVEL-2023-SCOT            VKRKTSSWKPPLPKAVSPAIAELRSIRKTQPLEGGTLNKGTTDGASTIGSGSLTGGTLKR      660
2019_E10_3_JHI-HISTORIC-SCOT        VKNKTSSWKSPLPKAVSPAIAKLRSIRKSQPLEGGTLKKDATDGVSSIGSGSLTGGTLKR      101
2021_E5_4_JHI-HISTORIC-SCOT         VKNKTSSWKPPLPKAVSPAIAKLRSIRKSQPLEGGTLKKDATDGVSSIGSGSLTGGTLKR      102
OK245432-HISTORIC-SCOT              VKNKTSSWKPPLSKAVSPAIAKLRSIRKSQPLEGGTLKKDATDGVSSIGSGSLTGGTLKR      660
                                    **.****** ** ********:******:********:*.:***.*:*************

2019_E11_3_JHI-NOVEL-2023-SCOT      KQT------------------------------------------------------      105
2019_E18_1_JHI-NOVEL-2023-SCOT      KQT------------------------------------------------------      105
2019_E20_4_JHI-NOVEL-2023-SCOT      KQTY-----------------------------------------------------      106
2021_E2_9_JHI-NOVEL-2023-SCOT       KQTY-----------------------------------------------------      106
2021_E3_10_JHI-NOVEL-2023-SCOT      KQTY-----------------------------------------------------      106
2022_E1_9_JHI-NOVEL-2023-SCOT       KQT------------------------------------------------------      105
2022_E2_2_JHI-NOVEL-2023-SCOT       KQT------------------------------------------------------      105
2022_E3_4_JHI-NOVEL-2023-SCOT       KQT------------------------------------------------------      105
PQ868077-NOVEL-2023-SCOT            KQTYEERLLQTLTTEQRLWYENLKKTDPGAATQWLFEYQPPPQVDRNIAEKPFQGRK      717
2019_E10_3_JHI-HISTORIC-SCOT        KVTV-----------------------------------------------------      105
2021_E5_4_JHI-HISTORIC-SCOT         KVT------------------------------------------------------      105
OK245432-HISTORIC-SCOT              KVTVEERLLQTLTTEQRLWYENLKKTNPPAATQWLYEYQPPPQVDRNMAEKPFQGRK      717
                                    * *
```
